# Citronellol-functionalized natural silica: a biogenic approach for antifungal and antibacterial material applications

**DOI:** 10.3389/fchem.2025.1535787

**Published:** 2025-01-30

**Authors:** Guillermo P. Lopez, Leyanet Barberia Roque, Katerine Igal, Erasmo Gámez Espinosa, Natalia Bellotti

**Affiliations:** ^1^ Laboratorio de Recubrimientos antimicrobianos, Centro de Investigación y Desarrollo en Tecnología de Pinturas y Recubrimientos-CIDEPINT, CICPBA-CONICET-UNLP, La Plata, Argentina; ^2^ Facultad de Ciencias Exactas-Universidad Nacional de La Plata-UNLP, Buenos Aires, Argentina; ^3^ Facultad de Ciencias Veterinarias-Universidad Nacional de La Plata-UNLP, Buenos Aires, Argentina; ^4^ Facultad de Ciencias Naturales y Museo-Universidad Nacional de La Plata-UNLP, Buenos Aires, Argentina

**Keywords:** diatomaceous earth, citronellol, functionalization, bioactive hybrids, terpenoid, antifungal, antibacterial, biocide

## Abstract

**Introduction:**

New bioactive hybrid materials to prevent biofilm-induced biodeterioration are a significant challenge in indoor environments, where contaminants from microbial films compromise structural integrity and contribute to air pollution, posing health risks from prolonged exposure to biological agents.

**Methods:**

For the first time, diatomaceous earth or diatomite (Dt) was functionalized with quaternary ammonium salt (QAS) and a biogenic compound, citronellol, to develop a bioactive hybrid material (Dt*QC). The hybrids obtained were characterized by thermogravimetric analysis (TGA), scanning electron microscopy (SEM), energy dispersive spectroscopy (EDS), and Fourier-transform infrared spectroscopy (FTIR). The antifungal and antibacterial activity were assessed by agar diffusion assay, and micro/macro-dilution tests.

**Results and Discussion:**

Characterization confirmed successful functionalization. TGA revealed organic contents of 50.9% with citronellol incorporation reaching 48.1%. SEM-EDS corroborated the incorporation of organic components. FTIR further verified the integration of functional groups while preserving the structural stability of the siliceous framework. Antimicrobial assays revealed a broader range of activity for Dt*QC. For bacterial strains, Dt*QC achieved a minimum inhibitory concentration (MIC) of 0.15 mg/mL against *Staphylococcus aureus* and demonstrated over 99.9% bacterial reduction, even at lower concentrations. This study highlights a novel approach to developing antimicrobial materials by functionalizing Dt with QAS and citronellol. Overall, these findings underscore the potential of Dt*QC as an advanced antimicrobial material for applications in coatings and preservation systems, offering a sustainable solution to prevent biodeterioration and microbial contamination.

## 1 Introduction

The degradation of material surfaces caused by biodeterioration poses significant challenges, particularly in indoor environments where individuals are frequently exposed to contaminants generated by biofilms (Gilbert and Stephens, 2018; Mu et al., 2021). Biofilms are complex communities of microorganisms with their extracellular materials, that adhere to surfaces and form protective matrices that enhance their resistance to conventional cleaning methods and antimicrobial agents (Harding et al., 2009). These microbial films compromise structural integrity and contribute to indoor pollution, posing health risks through prolonged contact with airborne biological agents (Kim et al., 2018). Adding to health concerns, frequent cleaning and the consequent material replacement led to increased environmental strain, economic losses, and higher energy consumption (Rauf and Crawford, 2015). Consequently, the development of sustainable and eco-friendly antimicrobial materials has become paramount. This approach aims to control biofilm formation effectively, extend the service life of materials, and mitigate the transmission of pathogens (Jeong et al., 2018; Kışla et al., 2023). Biogenic compounds such as terpenes, terpenoids, and phenolics found in plant essential oils exhibit a range of antimicrobial activities (Câmara et al., 2024). However, due to their characteristic volatility, these compounds are prone to evaporation when exposed to environmental conditions (Mahizan et al., 2019). Volatility refers to the inherent propensity of a substance to transition into the gaseous phase at relatively low temperatures. Although this contributes to their characteristic aroma, it limits their stability and diminishes their efficacy over time in applications demanding sustained antimicrobial activity. Therefore, their association or encapsulation must be studied to maintain functionality over extended periods and integrate them fully into everyday applications (Mahizan et al., 2019; de Oliveira et al., 2022). They can inhibit microbial growth by disrupting cell membranes, among other mechanisms (Yang et al., 2018; Mahizan et al., 2019). Integrating biogenic compounds into material matrices aims to enhance their efficacy by enabling the controlled release of active agents, thereby extending the antimicrobial service life of the materials (Himed et al., 2019; Oun et al., 2022). Although most studies are conducted by short-term *in vitro* tests, Wang et al. (2023) demonstrated the efficiency of cinnamaldehyde-loaded halloysite nanotubes through field experiments, which maintained their antimicrobial performance even after a year of exposure (Wang et al., 2023).

The present research explores the potential of a bioactive hybrid material by integrating natural silica (Dt) with citronellol, a biogenic compound to be applied as an antimicrobial filler in functional materials. Citronellol (3,7-dimethyloct-6-en-1-ol) is a natural monoterpenoid commonly extracted from essential oils of plants like *Cymbopogon citratus* (citronella grass) (Lanteri et al., 2020; Mesquita et al., 2021). Widely applied in fragrances, cosmetics, and personal care products, this compound has been approved as Generally Recognized as Safe (GRAS) by the U.S. Food and Drug Administration (FDA), confirming its suitability for applications with human contact (U.S. Food and Drug Administration-FDA, 2024). The GRAS status and its recognized bioactive properties position citronellol as a good candidate for environmentally friendly antimicrobial solutions.

Dt, a highly porous material with a modified surface structure, could be applied to integrate bioactive biogenic agents such as terpenoids (Lupu et al., 2020; Oun et al., 2022). The Dt used in this research (sourced from Argentina) is composed of natural silica obtained from the geological mineralization of deposits of unicellular algae, fossilized and sedimented at the bottom of large freshwater lakes over 70 million years ago (Fernández and Bellotti, 2017; Hasan et al., 2020b). This type of amorphous silica is characterized by its high porosity and intricate structure, which provides a substantial surface area. These properties make it ideal for various industrial applications, such as adsorbents in the chemical industry and filtration processes, allowing high efficiency in retaining impurities and fine particles (Akafu et al., 2019; Saidi and Hasan, 2022). The Dt morphological and chemical structure makes them advantageous in coatings and paints, where they function as gloss-control agents and provide mechanical strength and thermal stability (Kucuk et al., 2021; Alam et al., 2023). Due to its natural origin and extraction process, Dt also represents a low-cost and eco-friendly option compared to other additives based on synthetic silica encapsulates (Arreche et al., 2019; Mardones et al., 2019; Ogunsona et al., 2020). This is particularly crucial in antimicrobial material development where conventional antimicrobial agents or biocides frequently present significant risks. These risks include toxicity, environmental persistence, and the emergence of microbial resistance (Ribeiro et al., 2018; Dileep et al., 2020). As a result, there is a shift toward using naturally derived compounds and sustainable practices in antimicrobial material syntheses: bioactive montmorillonites hybrids with essential oils from, for example, oregano, thyme, and basil, for food packaging (Giannakas et al., 2017); metallic nanoparticles synthesized using aqueous extract of *Schinopsis balansae* and *Caesalpinia spinose* to antifungal coatings (Gámez-Espinosa et al., 2023); antimicrobial halloysite with carvacrol to antibacterial polyurethane films or food packaging (Hendessi et al., 2016; Alkan Tas et al., 2019). These eco-friendly alternatives align with green chemistry principles and favor more sustainable and safer applications for antimicrobial technologies.

The functionalization of materials with terpenoids like citronellol aligns with the principles of green chemistry and the increasing demand for sustainable antimicrobial solutions. Dt offers an adaptable and eco-friendly framework for incorporating organic antimicrobial agents, allowing for stable functionalization. By obtaining hybrids with natural silica, the high Dt surface pore structure can integrate bioactive agents and optimize their release, achieving sustained antimicrobial effects with reduced material waste and energy consumption (Moale et al., 2021). In a previous study, Dt was modified by QAS obtaining a bioactive hybrid to be applied as an antimicrobial filler (Fernández and Bellotti, 2017). Incorporating a second bioactive compound (citronellol) aims to broaden the antimicrobial spectrum and enhance its efficacy in functional material applications.

The present research aims to obtain a stable, functionalized natural silica material (Dt) modified by QAS and citronellol as an environmentally responsible, effective novel approach to prevent microbial growth materials. The QAS selected was hexadecyltrimethylammonium bromide ([CH_3_(CH_2_)_15_N(CH3)_3_]Br), a cationic surfactant highly soluble and considered safe (Fernández and Bellotti, 2017; Wang et al., 2024). The incorporation of the QAS into the silica framework would favor the subsequent organic antimicrobial agent incorporation such as citronellol. This innovative approach meets the need for effective, eco-friendly antimicrobial agents providing valuable insights into the potential of modified natural silica combined with a biogenic compound to be used as a bioactive filler for material preservation in indoor environments. The bioactivity of the Dt/QAS/citronellol hybrids was evaluated using standard microbiological assays (Fernández et al., 2020). Strains used were: *Chaetomium globosum* (KU936228), *Aspergillus fumigatus* (KU936230), *Penicillium commune* (KU936231), *Staphylococcus aureus* (ATCC 6538) and *Escherichia coli* (ATCC 11229). The fungal strains used were isolated from biodeteriorated materials in previous studies (Deyá and Bellotti, 2017; Gámez-Espinosa et al., 2020). The hybrids were characterized by SEM, energy dispersive spectrometry (EDS), FTIR, and TGA.

## 2 Materials and methods

### 2.1 Materials

The Dt used in this research is sourced from one of the primary extractive operations in the region, the deposits in Calingasta, San Juan, Argentina provided by DiatomiD. Quaternary ammonium salt (QAS), hexadecyltrimethylammonium bromide ([CH_3_(CH_2_)_15_N(CH_3_)_3_] Br), a cationic surfactant (Anedra). Citronellol was provided by Alfredo Francioni S. A. (Buenos Aires, Argentina).

### 2.2 Characterization of Dt

The composition of the Dt was studied by X-ray diffraction (XRD) performed in a Bruker D8 Advanced diffractometer, with an X-ray source with a Cu anode (Kα line = 1.54 Å), at 40 kV voltage, 30 mA current, a Ni filter, and SSD linear detector (LYNXEYE). The scan was conducted over the 3°–70° range in 2θ, with a step size of 0.02° and a dwell time per step of 0.5 s. The phase identification was performed by X´Pert HighScore de PANalytical software. Phase quantification and structural refinement were performed using Siroquant software based on the Rietveld method. This method involves fitting an experimental diffractogram with a theoretical one generated from a crystallographic model and experimental parameters to minimize the mathematical difference between observed and calculated intensities point by point across the diffraction pattern. It should be noted that this result does not account for the presence of amorphous phases or phases undetectable by XRD. The statistical error for the applied method on patterns is ±5 wt%. Morphological observations and elemental mapping were performed using a ZEISS Evo 10 scanning electron microscope (SEM-EDS).

### 2.3 Antimicrobial potentialities of citronellol

The antifungal and antibacterial activity of citronellol was assessed against strains of interest. In the case of fungi, *C. globosum* (KU936228), *A. fumigatus* (KU936230), *P. commune* (KU936231), that were isolated from biodeteriorated materials in previous research (Deyá and Bellotti, 2017; Roselli et al., 2020). Bacterial strains, *S. aureus* (ATCC 6538) and *E. coli* (ATCC 11229) are from the collection, and they have hygienic interest (Chung and Toh, 2014; Barberia-Roque et al., 2019). The agar diffusion method was carried out to assess the antimicrobial potentialities of the terpenoid (Fernández et al., 2020). In the case of the fungi, 15 mL of inoculated MEA was used, and 6 mm diameter paper discs were placed on each one. The plates were incubated at 28°C and after 48 h inhibition zone diameters (D) were measured. It was considered that samples with D < 6 mm exhibited no antifungal activity due to their invasive growth, whereas those with D ≥ 6 mm demonstrated activity. The bacterial strains were seeded by swabbing plates with LB agar culture medium using an inoculum of 10^6^ CFU/mL adjusted from diluted 0.5 Mc Farland (Lopez et al., 2023). The plates were incubated at 30°C and after 24 h inhibition zone diameters were registered, so samples with D = 6 mm exhibited no antibacterial activity whereas those with D > 6 mm proved activity. In all cases, three replicates were made.

### 2.4 Dt functionalization by citronellol

Initially, Dt was activated following the subsequent procedure: 10 g of Dt was mixed with 200 mL of a 2 M NaOH solution for 2 h at 100°C on a magnetic stirrer/hot plate (Fernández and Bellotti, 2017). The solid was separated by centrifugation and washed with distilled water (DW) to eliminate impurities. The pH was adjusted to around 10. The resulting solid (Dt*) was dried at 80°C. Subsequently, Dt* was dispersed in 200 mL of distilled water (DW) and combined with QAS in a 1:1 weight ratio. The mixture was stirred continuously for 24 h at room temperature. The resulting solid, labeled Dt*Q, was then separated by centrifugation, thoroughly washed with DW, and dried at 50°C to constant weight. Finally, citronellol was incorporated into Dt*Q by impregnating the material dropwise with the terpenoid at a 1:1 weight ratio (Pierattini et al., 2019). The hybrid obtained was labeled as Dt*QC and was dried in a Petri dish at 30°C for a week. After drying, the samples were weighed and transferred to amber-colored containers for storage.

### 2.5 Characterization of the functionalized Dt

Weight loss characteristics from each sample were assessed. TGA measurements were performed with a TA instrument Q50 TGA. The experimental conditions included a temperature range of 30°C–900°C, a heating rate of 5°C/min, and N_2_ atmosphere with a flow rate of 100 mL/min. Data analysis and curve generation were conducted using Universal Analysis 2000, Version 4.5A, Build 4.5.0.5. The FTIR spectra (4,000–400 cm^-1^) were performed using KBr pellet technique in a Perkin Elmer (Spectrum ONE) spectrometer. SEM analysis was performed using a Philips FEI Quanta 200 to examine the microstructure of samples. The solids were mounted on carbon tape and coated with gold for high-vacuum observation conditions. Semi-quantitative elemental composition was analyzed using EDS.

### 2.6 Antifungal activity

Firstly, to assess the antifungal potentialities of Dt, Dt*, Dt*Q, and Dt*QC agar diffusion assay was performed. In this case, the well variant of the assay was used, a 7 mm diameter well was made on each plate to be filled with 20 mg of each solid (Fernández et al., 2020). The same fungal strains and type of inoculum were used. Due to the characteristic fungi invasive growth, the observations were carried out after 48 h and the ninth day of incubation at 28°C. A stereoscopic microscope Leica S8 APO was used, and photographic records were obtained. Inhibition zone diameters were measured: D < 7 mm exhibited no antifungal activity, whereas those with D ≥ 7 mm showed activity. Three replicates were made for each sample studied.

To further investigate the antifungal activity of the citronellol hybrid (Dt*QC), the following macro-dilution plates assay was conducted to determine the minimum inhibitory concentration (MIC) (Lopez et al., 2023). Plates with MEA supplemented with different amounts of Dt*QC (5, 10, and 15 mg/mL) were prepared. In each case, 20 µL of the spore suspension (10^5^ spores/mL) was inoculated. The same strains employed in the previous bioassays were used. Plates were incubated at 28°C and the colony diameters were measured after 1 week of the assay.

### 2.7 Antibacterial activity

Antibacterial potentialities were assessed by agar well diffusion assay employing the same method and strains used previously. Wells of 7 mm were performed and filled with the studied solids in each plate with LB agar culture medium. After 24 h of incubation at 30°C inhibition zone D was registered in each case: D = 7 mm presented no antibacterial activity, whereas those with D > 7 mm showed activity. To further delve into the study of the antibacterial activity of the citronellol-based hybrid (Dt*QC), Minimum Inhibitory Concentration (MIC) was determined using a microdilution test in Luria-Bertani medium (LB) medium supplemented varying concentrations of Dt*QC (0.15, 0.3, 0.6, 1.2, 2.5, and 5 mg/mL) in a final volume of 3 mL (Oun et al., 2022). The samples were inoculated with 100 µL containing 10^6^ CFU/mL and incubated at 30°C with stirring (100 rpm) for 24 h. After that 100 µL was spread on LB agar plates and incubated for 24 h at 30°C for each sample. Three replicates were made for all samples studied.

On the other hand, the macro-dilution Erlenmeyer flasks assay was conducted using concentrations depending on the susceptibility of each bacterium obtained by the microdilution assay (Horue et al., 2024). The tests utilized 250 mL Erlenmeyer flasks, and bacterial enumeration was carried out using the viable plate count method. 1 mL of inoculum (10^6^ CFU/mL) was added to each flask, prepared by dilution from 0.5 McFarland overnight cultures of each bacterial strain. Dt*QC was suspended in fresh LB at concentrations tailored to the susceptibility of each strain: 3.5 mg/mL for *E. coli* and 0.10 mg/mL for *S. aureus*. Each inoculated flask contained a final volume of 100 mL. The flasks were incubated at 30°C under orbital shaking at 120 rpm for 24 h. After incubation, aliquots were serially diluted, plated over nutrient agar, and incubated at 30°C for 24 h. CFU was subsequently counted. All experiments were performed in triplicate to ensure reproducibility.

The relative means and the associated standard error for each experimental unit were calculated, considering the number of CFU as a discrete quantitative variable representing bacterial growth. The logarithms of results obtained were grouped in a bar chart. The percentage of inhibition (%I) was determined, considering growth controls without additives. All data processing was performed using the statistical package of Microsoft Excel.

## 3 Results and discussion

### 3.1 Characterization of the Dt

**FIGURE 1:**
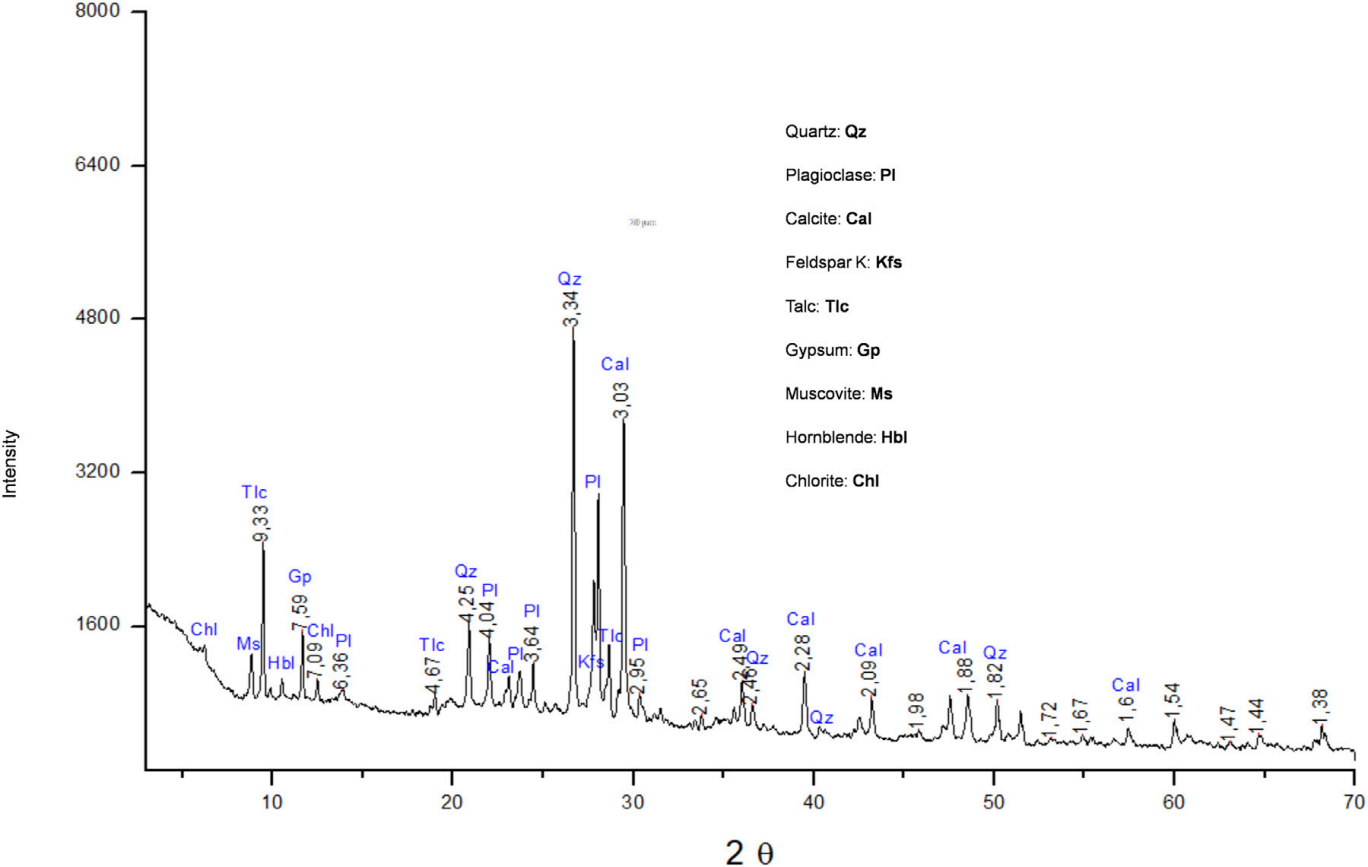
XRD diffractogram.

**FIGURE 2:**
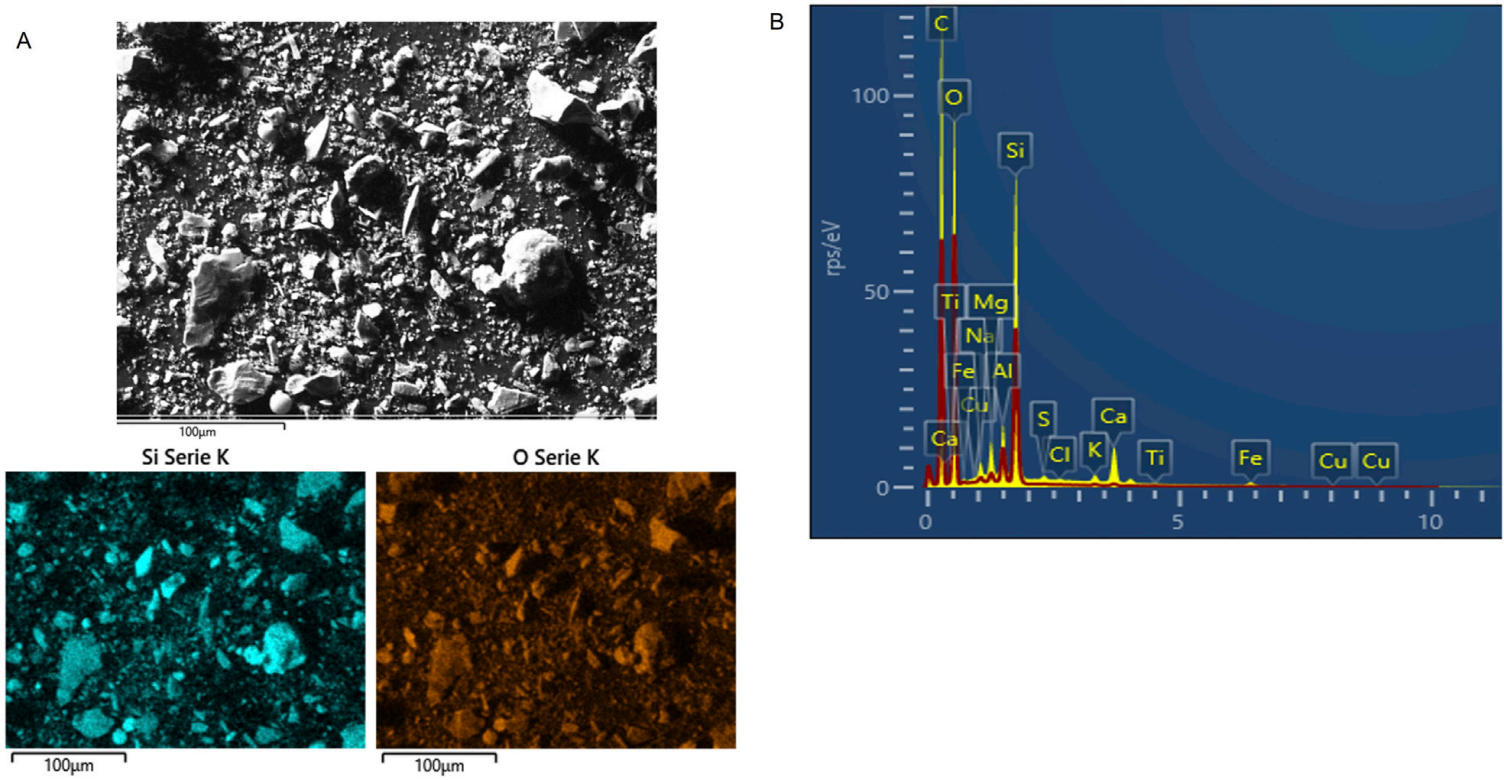
**(A)** SEM micrograph (magnification: 3,000x), Si and O mapping of the raw DE; **(B)** EDS spectrum.

XRD patterns are presented in Figure 1. The XRD analysis of raw Dt showed the presence of quartz (SiO_2_), plagioclase ((NaCa) (SiAl)_3_O_8_), feldspar K (KAlSi_3_O_8_), and calcite (CaCO_3_) as majority phases. From observations carried out by SEM the Dt microstructure presented mostly parts of the diatoms, and therefore complete preserved structures were not found. In the micrograph of Figure 2A, the morphological heterogeneity of the sample can be observed. Superficial analysis by EDS corroborated the majority presence of Si and O. The mapping of Si and O over the sample can be observed in Figure 2A, which revealed a homogeneous distribution of this element. Minor content of other elements (Ca, Al, Mg, Fe, Na, and K) was detected as shown in Figure 2B. The C detected mostly was attributed to the carbon tape used to mount the samples.

### 3.2 Antimicrobial potentialities of citronellol

**FIGURE 3:**
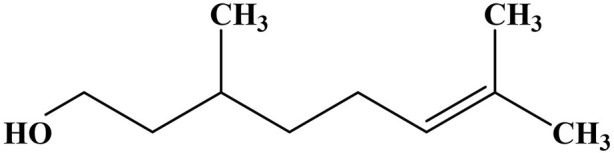
Chemical structures of the Citronellol.

The chemical structure of citronellol can be seen in Figure 3. Table 1 presents the results of the agar diffusion assay, including the average inhibition zone diameters (D) and their corresponding standard deviations. Citronellol was active against fungal and bacterial strains with D higher than 20 mm. *Chaetomium globosum* was shown to be the more sensitive one with D = 75 mm while *A. fumigatus* was the most resistant with 26 mm. In the case of bacteria, citronellol was active against both strains but *E. coli* was the more resistant. Photographic registers are shown in Supplementary Materials Figure 1. Therefore, this terpenoid is promising due to the broad spectrum of antimicrobial activity demonstrated against the target microorganisms. It is important to note that the fungal and bacterial strains used in this study were selected for their relevance to material biodeterioration and their significant impact on health, highlighting the practical implications of the antimicrobial assessment for both material preservation and public health (Adan and Samson, 2011; Chung and Toh, 2014; Gilbert and Stephens, 2018).

**TABLE 1:** Agar diffusion assay: inhibition zone diameters (mm).

Strains	Citronellol	Dt*	Dt*Q	Dt*QC
*A. fumigatus*	26 ± 2	<7	18 ± 1	37 ± 1
*C. globosum*	75 ± 4	<7	20 ± 3	87 ± 11
*P. commune*	36 ± 3	<7	20 ± 1	37 ± 4
*E. coli*	20 ± 1	7	7	19 ± 1
*S. aureus*	29 ± 3	7	25 ± 1	38 ± 2

### 3.3 Characterization of the functionalized Dt

**FIGURE 4:**
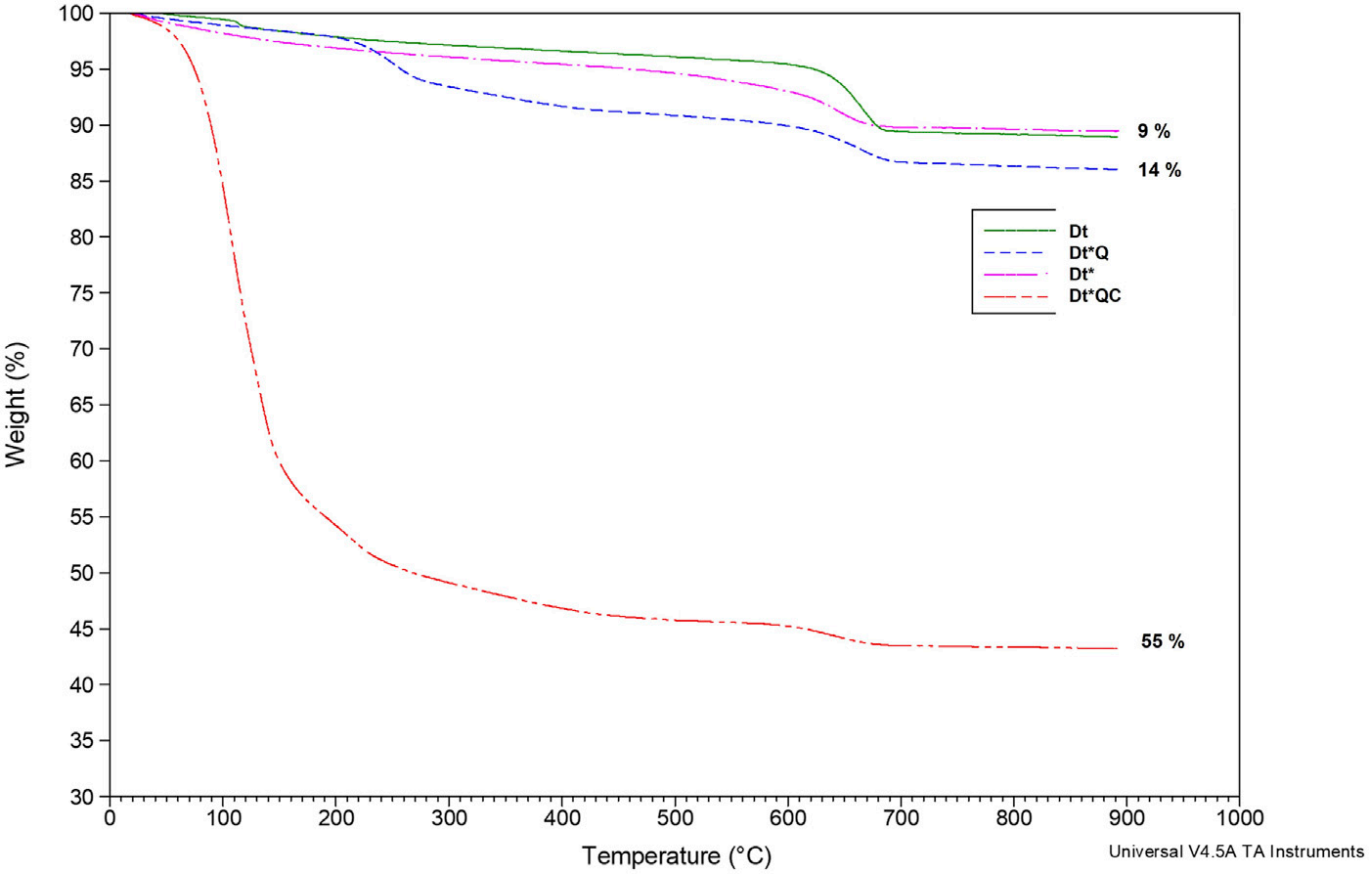
TGA: Dt, Dt*, Dt*Q and Dt*QC.

Figure 4 illustrates the TGA curves showing the weight loss results. Both Dt and Dt* recorded a weight loss of 9%. Dt*Q and Dt*QC showed weight losses of 14% and 55%, respectively. Two significant episodes of weight loss were observed. The first occurred between 100°C and 300°C, which may be associated with dehydration of water adhering to the Dt structure, and the second occurred between 600°C and 700°C, corresponding to the total removal of QAS and citronellol, in the respective samples, being the organic fraction corresponding with 5.4% in Dt*Q and 50.9% in Dt*QC. TGA analysis confirmed the successful functionalization of Dt* by QAS and the incorporation of the citronellol. The amount of incorporated terpenoid was substantial, 48.1% of the Dt*QC. This represents a significant improvement compared to previous studies with clays, where terpene incorporation did not exceed 20% (Nagy et al., 2013; Fernández et al., 2020). The higher incorporation rate may enhance the antimicrobial efficacy and stability over time, potentially broadening its applications in fields requiring sustained antimicrobial activity, such as coatings and preservation treatments.

**FIGURE 5:**
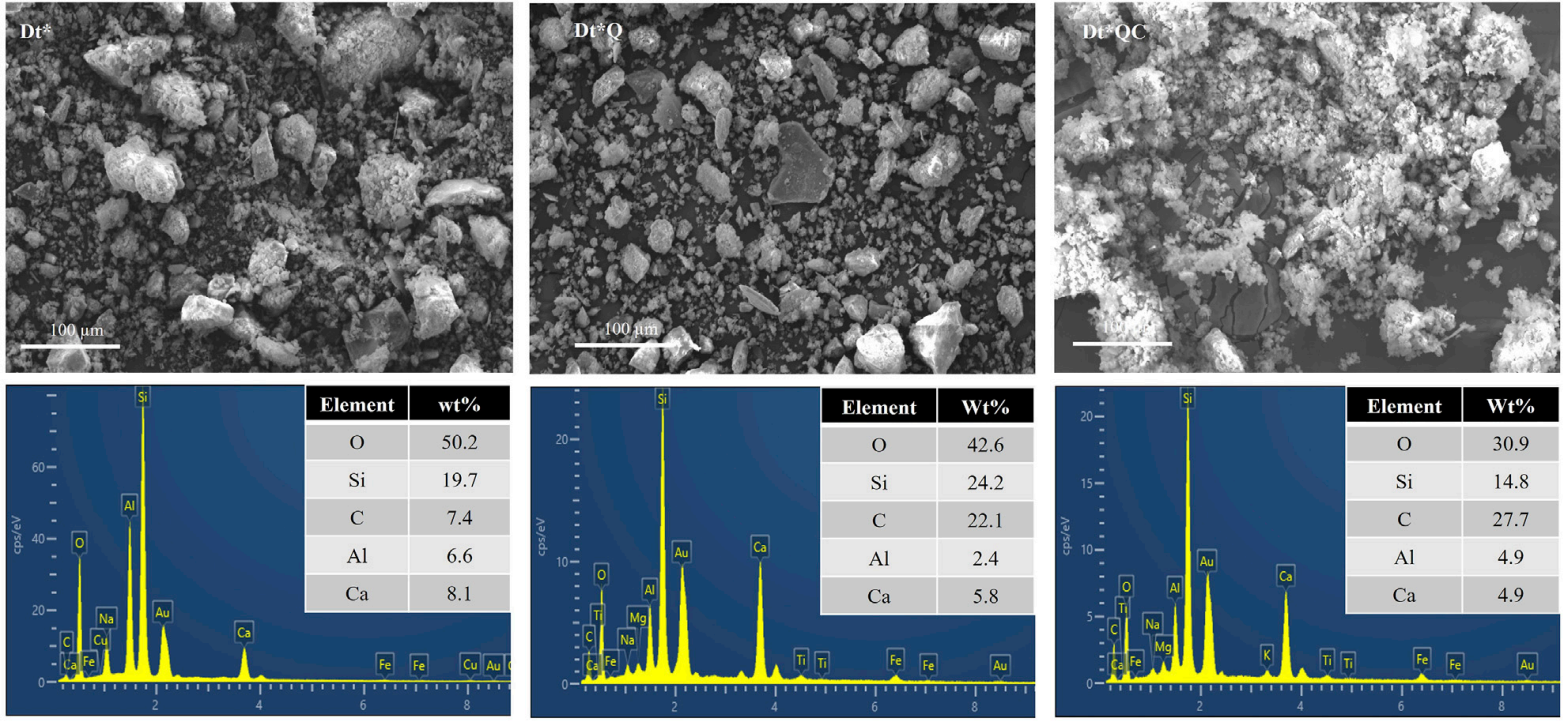
SEM micrographs (magnification ×500) and EDS elemental surface analysis: Dt*, Dt*Q, and Dt*QC.

SEM micrographs of Dt*, Dt*Q, and Dt*QC are shown in Figure 5. The micrograph from Dt* reveals fragmented, porous, and angular structures typical of diatomaceous earth. EDS superficial analysis is presented in Figure 5. Elements primarily consist of O (50.2%), Si (19.7%), and Ca (8.1%), along with minor content of Al and C to Dt*. In all cases, the wt% is the average of three determinations on each sample after EDS analysis. Following QAS integration, Dt*Q exhibits a more granular morphology. EDS analysis indicates a decrease in O content (42.6%) and an increase in Si (24.2%) and C (22.1%) this last one is associated with the QAS organic integration. The citronellol addition resulted in a denser structure, with visible agglomeration and less-defined particle boundaries. EDS analysis of Dt*QC shows a further increase in C content (27.7%) and a marked decrease in O (30.9%), suggesting organic incorporation. The lower percentage of organic (5.4%) in Dt*Q compared to Dt*QC (50.8%) as determined by TGA, highlights a notable difference when contrasted with the surface wt% of C obtained from EDS analysis. These differences may suggest that the QAS is primarily distributed at the surface level in Dt*Q and citronellol with lower molecular weight achieves a more uniform distribution both on the surface and within the internal structures. The morphological changes and elemental shifts observed in Dt*Q and Dt*QC indicate that QAS and citronellol are effectively incorporated into the diatomaceous structure. The further addition of citronellol in Dt*QC builds upon this, as evidenced by the morphological densification and increased C content, suggesting citronellol association and retention within the matrix. The TGA findings support this integration, where the substantial weight loss in Dt*QC is primarily due to the citronellol component, reinforcing the material potential for antimicrobial applications.

**FIGURE 6:**
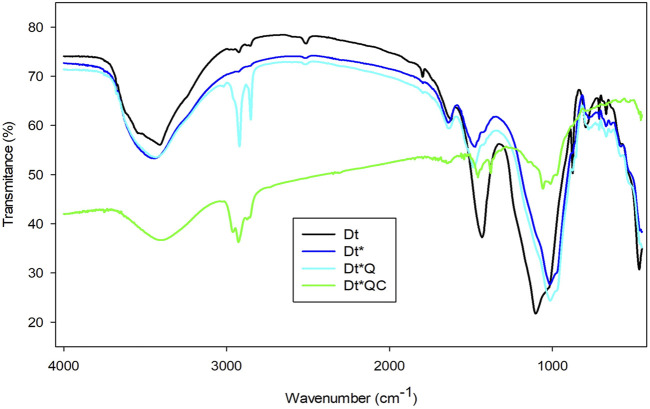
FTIR spectra: Dt, Dt*, Dt*Q, and Dt*QC.

The FTIR spectra of Dt, Dt*, DtQ, and Dt*QC are presented in Figure 6. The broad band observed around 3,300 cm⁻^1^ in all samples suggests the presence of hydroxyl (OH) groups (Salih and Ghosh, 2018). This band is more intense in the samples after functionalization indicating the corresponding surface modification. The increased OH groups exposed after alkaline activation would increase hydrophilicity and prepare Dt* for the QAS treatment in an aqueous solution. The peaks around 2,900 cm⁻^1^, noticeable in the functionalized samples (Dt*Q and Dt*QC), are associated with C-H stretching vibrations (Oun et al., 2022). This indicates the presence of organic components, such as QAS and citronellol, integrated into the silica. The intensity of the peaks correlates with the amount of organic functionalization. Raw Dt shows an intense peak at 1,435 cm⁻^1^ attributed to Si-OH vibrations. After alkaline activation, this peak shifts to 1,480 cm⁻^1^ in Dt* and decreases in intensity, suggesting surface structural changes due to deprotonation or rearrangement on the material surface. The peak at 1,381 cm⁻^1^ only observed in Dt*QC may be associated with the characteristic vibrations of C-H bonds from methyl or methylene groups, typical in organic compounds such as citronellol (Cooper James W, 1982). The prominent peak around 1,000 cm⁻^1^ corresponds to the Si-O stretching mode, a characteristic feature of silica (Hasan et al., 2020a). This peak remains relatively consistent across all samples, indicating that the silica framework remains intact despite functionalization. This stability is important, as it suggests that the functionalization process does not compromise the structural integrity of the siliceous material. The widening and slight shifts in the O-H and Si-O bands suggest interactions between the silica surface and the functional groups in QAS and citronellol (Oun et al., 2022). C-H and O-H peaks alongside the stable Si-O peak indicate a balance between organic functionalization and structural stability. This could enhance the material functionality for the application in antimicrobial coatings or filtration systems.

### 3.4 Antifungal activity

**FIGURE 7:**
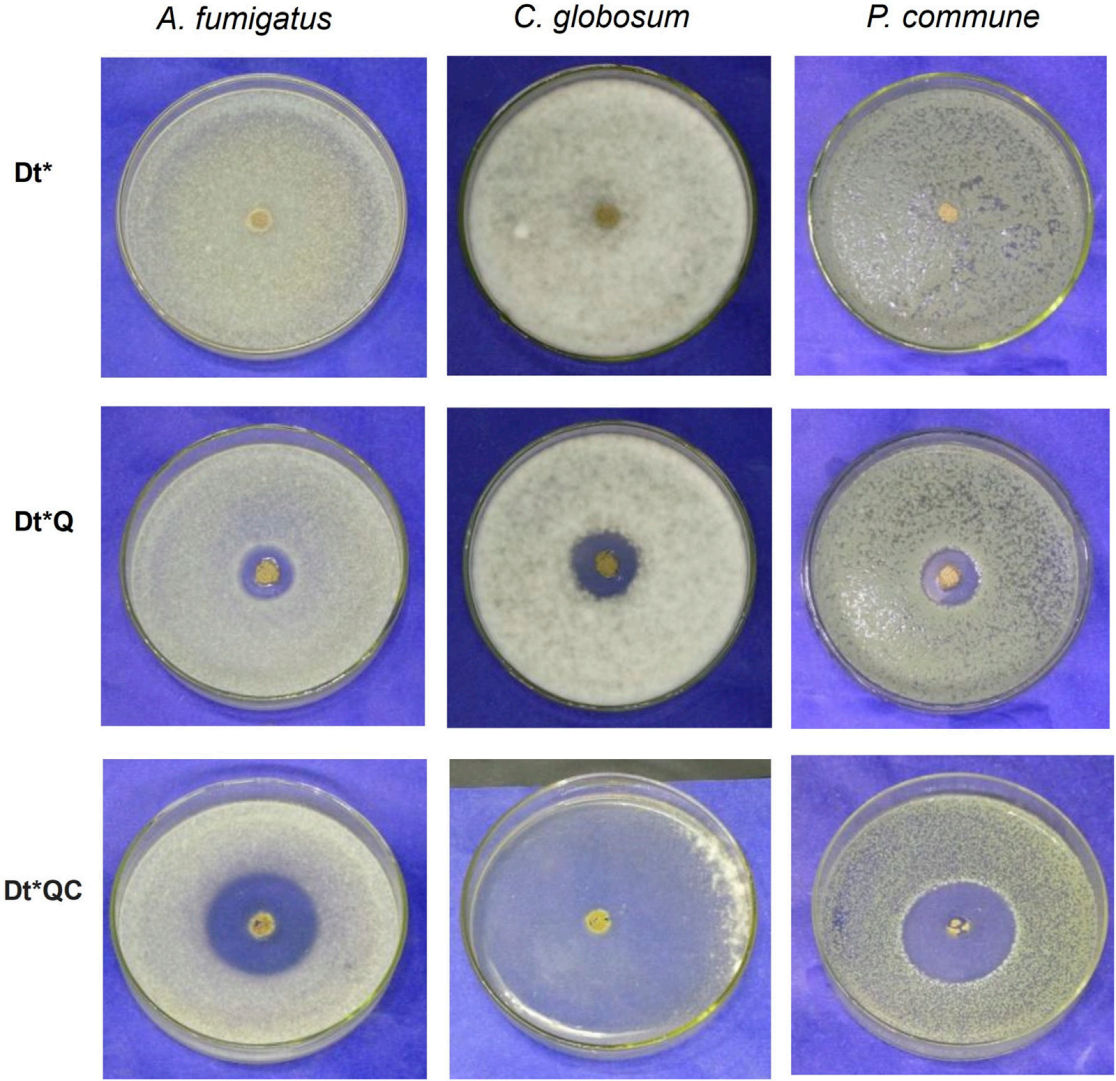
Agar well diffusion assay: Dt*, Dt*Q, and Dt*QC against *Aspergillus fumigatus*, *C. globosum*, and *P. commune*.

The diffusion assay provides insight into the antimicrobial activity of the Dt modified with QAS and citronellol. Table 1 shows inhibition zone D for each case. Figure 7 presents photographic records of the agar diffusion assay after 48 h of incubation. The observed inhibition zones around the samples suggest varying levels of antimicrobial efficiency. Dt* showed fungal growth over the samples indicating an absence of inherent antifungal properties with D values below 7 mm. The same results were registered with Dt. When Dt* was modified with QAS alone (Dt*Q), there was a noticeable increase in activity against all fungal strains, as indicated by D ranging from 18–20 mm. The Dt*QC that combines both, QAS and citronellol, exhibited the highest inhibition zones across all strains. For instance, the D against *A. fumigatus* reached 37 mm, compared to 18 mm for Dt*Q. These enhanced diameters were especially pronounced against *C. globosum* being 87 mm, compared to 20 mm for Dt*Q. The substantial difference would be due to a synergistic effect between the QAS and citronellol, where the cationic sites provide initial membrane disruption, allowing citronellol to penetrate microbial cells more effectively and inhibit growth. The samples were evaluated after 9 days to confirm if the antifungal activity was maintained over time. The stereoscopic microscope images are shown in the Supplementary Materials Figure 2. The hybrids with QAS and citronellol were achieved to maintain the antifungal activity with higher inhibition zones. In the Dt* controls a heavy sporulation rate can be observed with all strains.

The macro-dilution plates test showed total inhibition against the three fungal strains (*A. fumigatus*, *C. globosum*, and *P. commune*) with the lower concentration of Dt*QC used (5 mg/mL). The antifungal activity of citronellol is attributed to several mechanisms. Terpenes can interact with microbial cell membranes primarily due to their nonpolar alkyl chain, which allows them to embed within the lipid bilayer (Mahizan et al., 2019). This integration disrupts the structural integrity of the membrane, compromising its selective permeability and causing an imbalance in ion exchange and nutrient transport (Zhang et al., 2023). In the case of fungi, they can inhibit the synthesis of ergosterol, an essential component of fungal cell membranes, weakening the membrane and compromising their viability (Câmara et al., 2024; Milovanovic et al., 2024). Additionally, terpenes may inhibit key fungal enzymes, obstructing critical metabolic processes. They can induce oxidative stress by generating reactive oxygen species within fungal cells, causing oxidative damage to lipids, proteins, and nucleic acids, ultimately leading to cell death (Rolim et al., 2024).

Furthermore, studies have demonstrated the effectiveness of QAS in inhibiting fungal growth, including against *Candida albicans*, a common fungal pathogen (Vieira and Carmona-Ribeiro, 2006). This inhibition mechanism involves the adsorption of QAS onto the fungal cell surface, leading to cell viability and membrane integrity alteration. Specifically, the QAS adsorption produced a significant reduction in cell viability, with concentrations as low as 0.3 mM causing a 50% decrease in survival. Furthermore, QAS-induced changes in cell electrophoretic mobility suggest that the surfactant interacts with the fungal cell membrane, disrupting its normal function (Vieira and Carmona-Ribeiro, 2006). The antimicrobial activity of the QAS structure originates from its positively charged group that adsorbs on the generally negatively charged membrane surfaces which allows then hydrophobic interaction through its alkyl chains and the phospholipid bilayer destabilizing them and causing the leakage of intracellular material which can lead to cell death (Wang et al., 2024). Another study investigated the use of QAS in developing a bio-based antifungal nano-material by coating it onto the surface of cellulose nanocrystals (Xiang et al., 2019). This composite showed enhanced antifungal activity against *Phytophthora capsici*, a plant pathogen. The increased efficacy was attributed to the higher local concentration of QAS on the surface which facilitated more effective interaction with the fungal cell membrane, leading to increased permeability and cell death (Xiang et al., 2019).

### 3.5 Antibacterial activity

**FIGURE 8:**
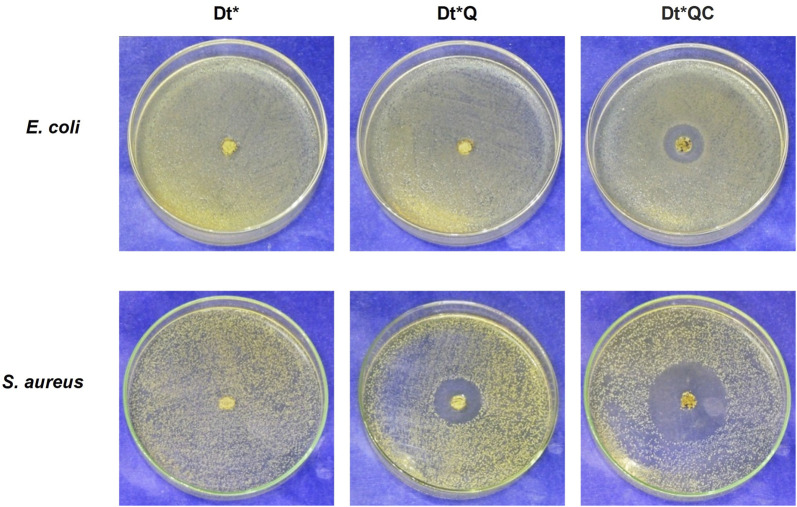
Agar well diffusion assay: Dt*, Dt*Q, and Dt*QC against *Escherichia coli* and *S. aureus*.

Table 1 shows the diffusion assay results for each sample and Figure 8 provides photographic records after 24 h of incubation. Dt* did not exhibit any antimicrobial activity against either bacterial strain tested. In contrast, Dt*Q showed activity exclusively against *S. aureus*. Dt*QC demonstrated activity against both bacterial strains, with 19 mm and 38 mm inhibition zones, respectively. The results suggest that Dt* modification significantly enhanced its antimicrobial efficiency, particularly in the case of Dt*QC. Once again, *E. coli* demonstrated the highest resistance among the tested strains. In the microdilution test (liquid medium), the MIC for Dt*QC exceeded 5 mg/mL; however, while complete inhibition was not achieved at this concentration, the degree of inhibition was notably high. In contrast, for *S. aureus*, the MIC was significantly lower, at 0.15 mg/mL. The partial solubility of citronellol in aqueous media, compared to the high solubility of QAS, may influence the bioactivity of the terpenoid in liquid systems, particularly against more resistant bacteria like *E. coli* (Guimarães et al., 2019). Once again, *E. coli* proved to be the most resistant strain. The differential response to Dt*QC between the two strains underscores the importance of differences in cell wall structure in determining the susceptibility of bacteria to antimicrobial agents. Gram-positive bacteria like *S. aureus*, which lack an outer membrane, are generally more vulnerable to compounds that can disrupt cell walls or membranes, this is consistent with the inhibitory effect seen at lower Dt*QC concentrations (Xu et al., 2023). On the other hand, the presence of the outer membrane in Gram-negative bacteria like *E. coli* reduces permeability, decreasing the efficiency of Dt*QC with a higher MIC (Mahizan et al., 2019).

**FIGURE 9:**
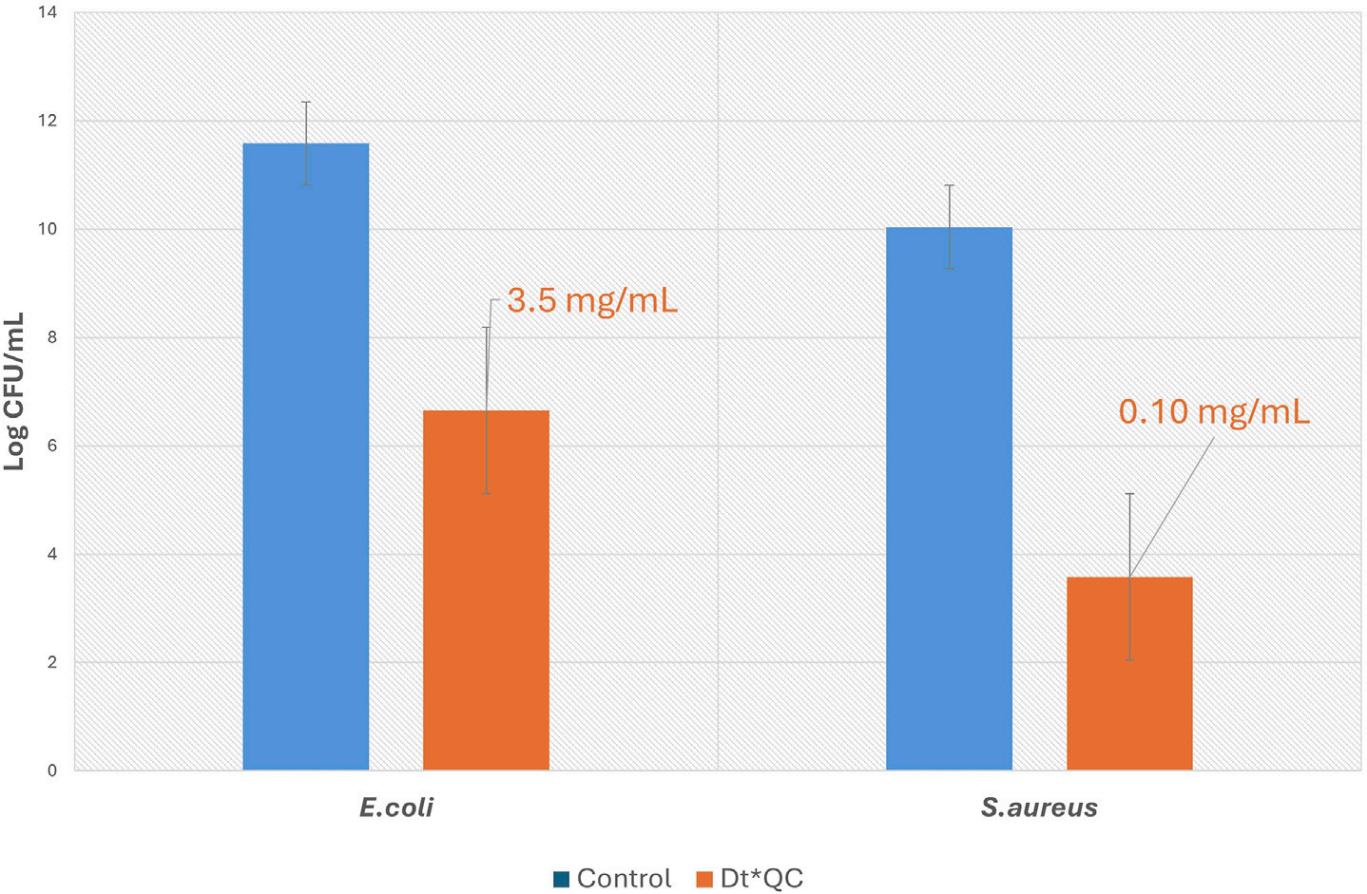
The bar graph illustrates the logarithms of CFU for *Escherichia coli* and *S. aureus* with and without Dt*QC. The control bars correspond with the culture medium without Dt*QC.

The results obtained by macro-dilution assay are presented in Figure 9. The mean of CFU/mL for *E. coli* was 3.80.10^11^ for the control, while in the medium supplemented with Dt*QC, the mean was 4.48.10^6^. In control, *S. aureus* grew on average 1.09.10^10^ CFU/mL, while in the culture medium supplemented with Dt*QC 3.80.10^3^ CFU/mL was counted. The bar graph shows the reduction in bacterial growth by more than five orders caused by Dt*QC addition to the culture medium against both bacteria. Moreover, the trend observed in previous experiments was maintained, and *S. aureus* was more sensitive than *E. coli* to Dt*QC, even at lower concentrations. The inhibition percentage provided for Dt*QC exceeded 99.9% against both bacteria. Photograph registers from the macro-dilution test are shown in the Supplementary Materials Figure 3.

These findings significantly contribute to advancing material science and antimicrobial technologies. The successful functionalization of diatomaceous earth (Dt) with QAS and citronellol (Dt*QC) demonstrates an efficient pathway for obtaining materials with broad-spectrum antimicrobial activity. This innovation could play a role in addressing challenges to control material biodeterioration by integrating these hybrids in coatings formulations, or food packaging among other applications.

Future research should focus on scaling up the synthesis process while optimizing the functionalization to enhance the material stability and performance under field-trial conditions. Additionally, expanding the scope of this technology to target specific pathogens or integrating synergistic antimicrobial agents could increase its potential for tailored applications. The high incorporation efficiency of citronellol and its sustained activity in this study suggest that Dt*QC could be used to formulate hygienic paints and coatings. These advancements contribute to scientific knowledge but also offer practical solutions to pressing global issues related to microbial resistance and material degradation.

## 4 Conclusion

The present research successfully developed a novelty bioactive hybrid material by functionalization of diatomaceous earth with a quaternary ammonium salt and citronellol (Dt*QC) offering a sustainable and effective approach to mitigate biodeterioration and microbial contamination in indoor environments. The diatomaceous silica matrix provided an efficient QAS and citronellol functionalization framework, enabling sustained release and enhanced antimicrobial activity.

The results demonstrated that the functionalization process preserved the structural integrity of the silica, as confirmed by FTIR and TGA analyses while introducing significant organic functionality. The antimicrobial assessments highlighted the synergistic effect of QAS and citronellol, resulting in broad-spectrum activity against both fungal and bacterial strains. Notably, Dt*QC exhibited superior antifungal and antibacterial activity, maintaining efficacy over time, a critical factor for prolonged material preservation.

These findings underscore the potential of hybrid materials based on natural silicas and biogenic compounds like citronellol as eco-friendly antimicrobial solutions. The hybrid ability to prevent microbial colonization, prolong material durability, and align with green chemistry principles is a promising alternative to traditional chemical biocides. Future research should evaluate its performance in applications, such as coatings or filtration systems.

## Data Availability

The original contributions presented in the study are included in the article/Supplementary Material, further inquiries can be directed to the corresponding author.
