# Supplementary figures and images for: Citronellol-functionalized natural silica: a biogenic approach for antifungal and antibacterial material applications
Source: Front Chem. 2025 Jan 30;13:1535787. doi: 10.3389/fchem.2025.1535787 (PMC11821633; doi:10.3389/fchem.2025.1535787)

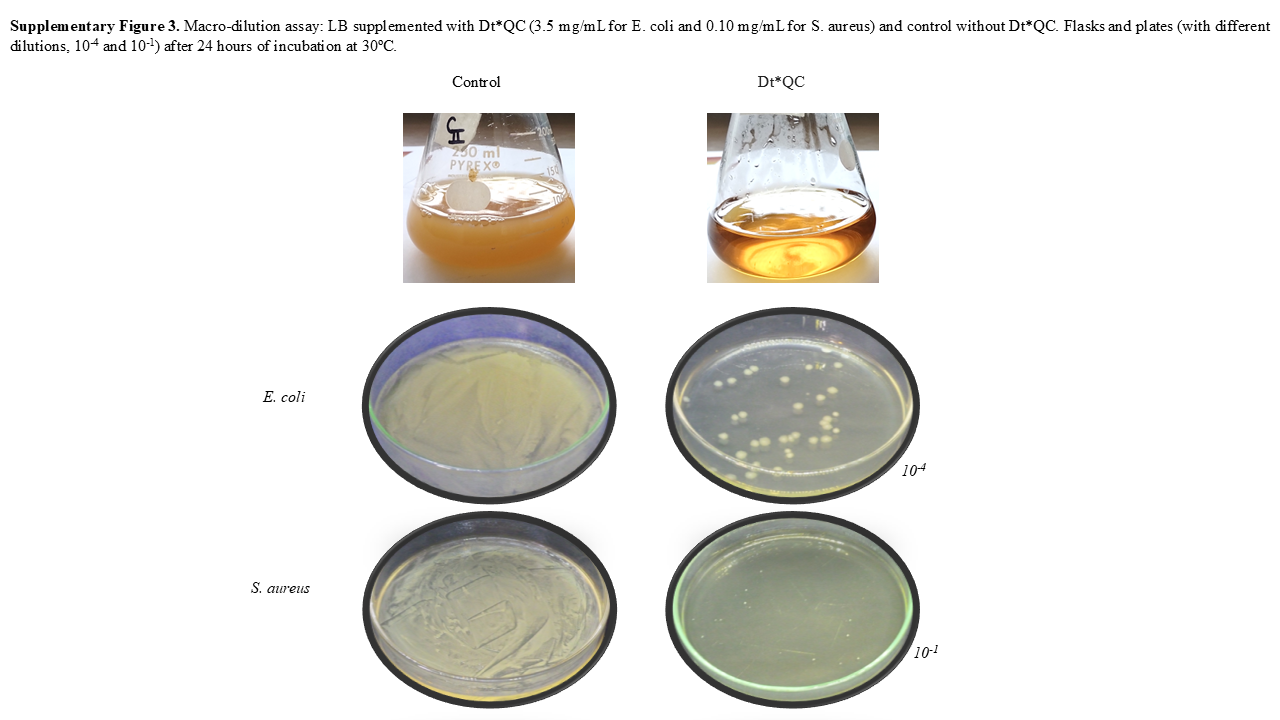

Supplement: Supplementary file 1 [file Image3.tif]

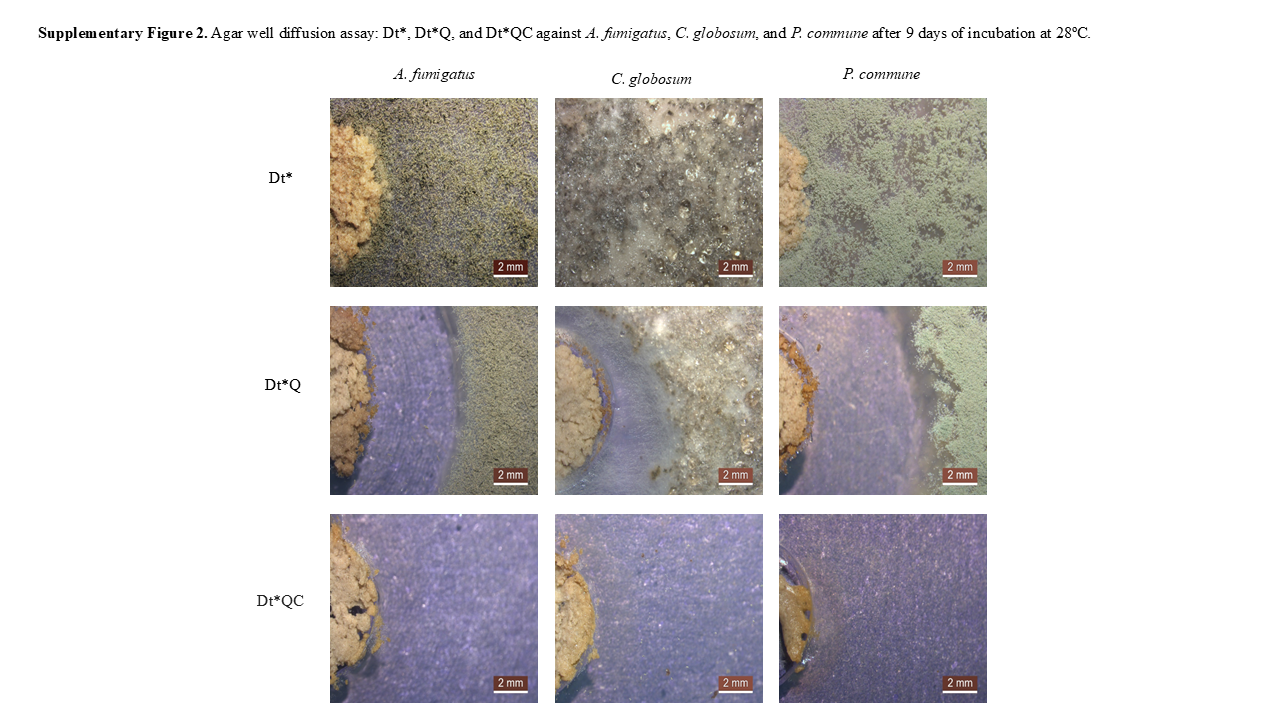

Supplement: Supplementary file 2 [file Image2.tif]

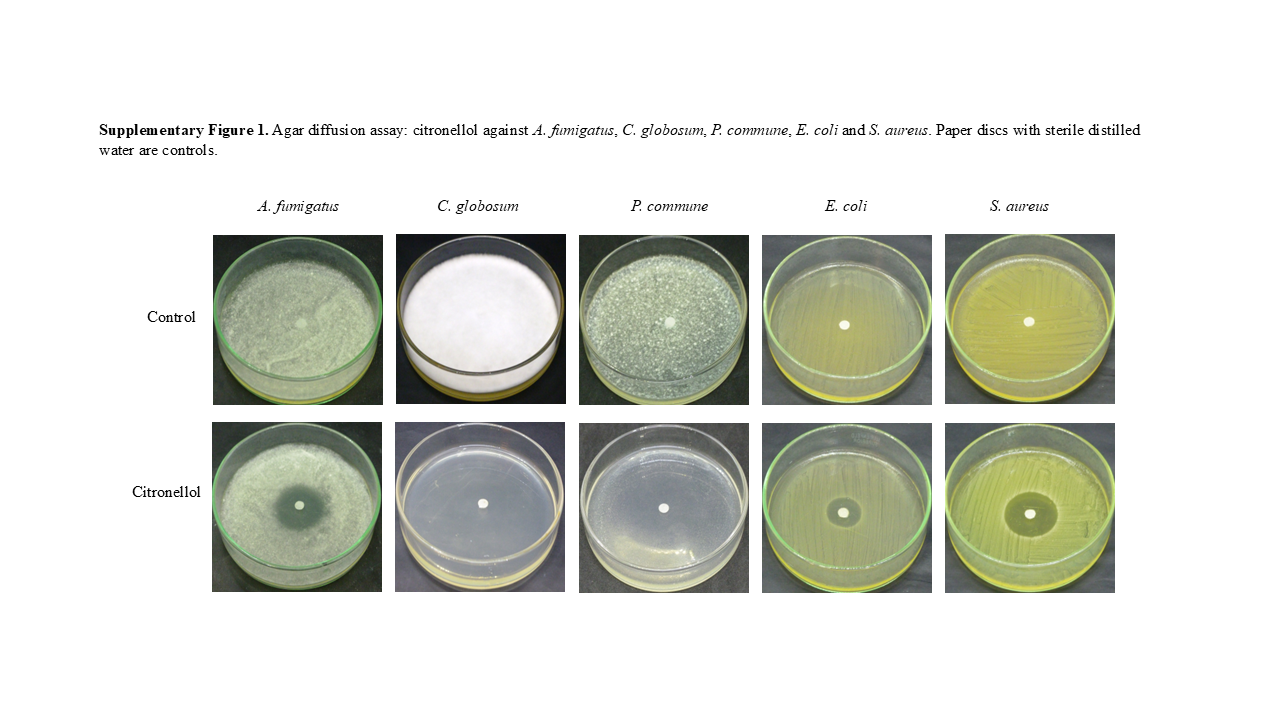

Supplement: Supplementary file 3 [file Image1.tif]
